# The effectiveness of motorised lumbar traction in the management of LBP with lumbo sacral nerve root involvement: a feasibility study

**DOI:** 10.1186/1471-2474-8-118

**Published:** 2007-11-29

**Authors:** Annette A Harte, George D Baxter, Jacqueline H Gracey

**Affiliations:** 1 Health Rehabilitation Sciences Research Institute, University of Ulster, Newtownabbey, Northern Ireland; 2 School of Physiotherapy, University of Otago, Dunedin, New Zealand

## Abstract

**Background:**

Traction is commonly used for the treatment of low back pain (LBP), predominately with nerve root involvement; however its benefits remain to be established. The aim of this study was to test the feasibility of a pragmatic randomized controlled trial to compare the difference between two treatment protocols (manual therapy, exercise and advice, with or without traction) in the management of acute/sub acute LBP with 'nerve root' involvement.

**Methods:**

30 LBP patients with nerve root pain were recruited and randomly assigned to one of two treatment groups. Primary outcome measures were the: McGill pain questionnaire, Roland Morris disability questionnaire, and the SF36 Questionnaire; recorded at baseline, discharge, 3 and 6 months post-discharge.

**Results:**

27 patients completed treatment with a loss of another four patients at follow up. Intention to treat analysis demonstrated an improvement in all outcomes at follow up points but there appeared to be little difference between the groups.

**Conclusion:**

This study has shown that a trial recruiting patients with 'nerve root' problems is feasible. Further research based upon a fully powered trial is required to ascertain if the addition of traction has any benefit in the management of these patients.

**Trial Registration:**

Registration number: ISRCTN78417198

## Background

There is ongoing confusion surrounding the use of traction in the management of low back pain (LBP), with differences between recommendations in the UK, New Zealand, Denmark and the USA clinical guidelines [1]. This is further confounded by a recent Cochrane systematic review which concluded that 'traction probably is not effective,' however, the authors also noted that '*we lack strong, consistent evidence regarding the use of traction due to the lack of high quality studies, the heterogeneity of study populations, and lack of power. Any future research should distinguish between symptom pattern and duration and should be carried out according to the highest methodological standard to avoid bias *[2].'

Despite such recommendations, traction continues to be commonly used by physiotherapists in the management of LBP; a recent UK-wide survey indicated that 41% of therapists used traction with 5% of LBP patients, who almost exclusively presented with 'nerve root' problems [3]. Between 3 – 10% of LBP sufferers will experience 'sciatica' or 'nerve root' pain, with or without neurological signs [4-6] with 90% recovering, but a further 10% requiring surgery [6]. Guidelines highlight this small group of patients in their triage system with the implication that this group of patients may be more severe, slower to recover, and may require specialist referral when compared to 'simple' LBP [5,7-10]. Effective management of this group of patients is therefore essential to limit costly onward referral and surgery that may result.

This study was designed to assess the feasibility of a pragmatic randomized controlled trial (RCT) designed to examine the effectiveness of traction with this subgroup of LBP, employing treatment parameters indicated by clinical practice and expert opinion [3]. As manual therapy is often used in conjunction with traction in the management of 'nerve root' problems, this study compared the addition of traction to a manual therapy treatment protocol (manual therapy, exercise and advice, with or without traction).

The specific objectives of this study were to ascertain the feasibility of the study protocol, in particular the screening and adequate recruitment of 'nerve root' patients.

## Methods

Ethical approval was granted by the Research Ethical Committee of the University of Ulster. This multicentred, pragmatic randomized controlled trial was set in three physiotherapy departments in the Down Lisburn Health and Social Care Trust, Northern Ireland. General Practitioners in this catchment area were contacted to ensure early referral to physiotherapy of LBP patients with nerve root involvement.

### Subjects were included if they fulfilled the following criteria

(i) Aged 18–65 years of age (male and female), presenting with acute/sub-acute LBP with accompanying radiculopathy;

(ii) Radiculopathy or 'nerve root' was identified by the presence of:

Dermatomal pain distribution radiating below the knee (one or both limbs), of a sharp/severe quality, often worse in the leg than back (leg pain threshold of 3/10 VAS).

With at least one of the following signs and symptoms:

(a) Pins and needles in the distal dermatome (where this was present patients with leg pain were accepted even if not extending below the knee);

(b) Increased pain in the leg on coughing, sneezing or straining;

(c) Neurological deficit i.e. decreased muscle strength/sensory loss/reflex loss;

(d) Positive straight leg raise test i.e. limb symptoms reproduced on SLR test below 90 degrees [5,8,10-12];

(iii) Acute/sub acute LBP, defined as LBP of less than 12 weeks duration [5,7], or a recurrent episode with a pain free period of at least three months prior to the onset of this episode. Only one study has considered recovery rates with 'sciatica' [13] and reported that both back and leg pain decreased, on average, by 69%, and disability decreased by 57% within one month from onset. Current physiotherapy practice would suggest that treatment begins as soon as possible; therefore patients were accepted after 4 weeks of onset of leg pain;

(iv) Able to attend for physiotherapy 2–3 times a week for 4–6 weeks;

(v) Patients were literate with English as their first language.

### Subjects were excluded if they presented with

(i) Previous spinal surgery;

(ii) Formal therapeutic or medical intervention within the last three months (eg epidural injection, facet joint block, physiotherapy etc);

(iii) Co-existing conditions (anklyosing spondolytitis, rheumatoid arthritis, spinal stenosis (diagnosed), spondolythesis, recent spinal fracture, spinal tumor or a patient where secondary metastases was suspected);

(iv) Concomitant severe medical problem preventing participation in the trial (cardiac condition, respiratory conditions, neurological disorder or organ disease);

(v) Long term oral steroid intake (due to the risk of osteoporosis);

(vi) Current anti-coagulant therapy or blood clotting disorders;

(vii) Pregnancy;

(viii) History of major psychiatric illness;

(ix) Roland Morris disability questionnaire score of below 4, and/or a VAS score of less than 3 on a 10 point scale for leg pain (to avoid floor effects).

### Research design

The research design was an investigator-blinded pragmatic RCT with two active treatment arms.

Two physiotherapists were appointed in each of the three research sites: these were responsible for the initial screening of incoming referrals, onward referral of patients to the research therapist, and treatment of patients entered into the trial.

**Figure 1:**
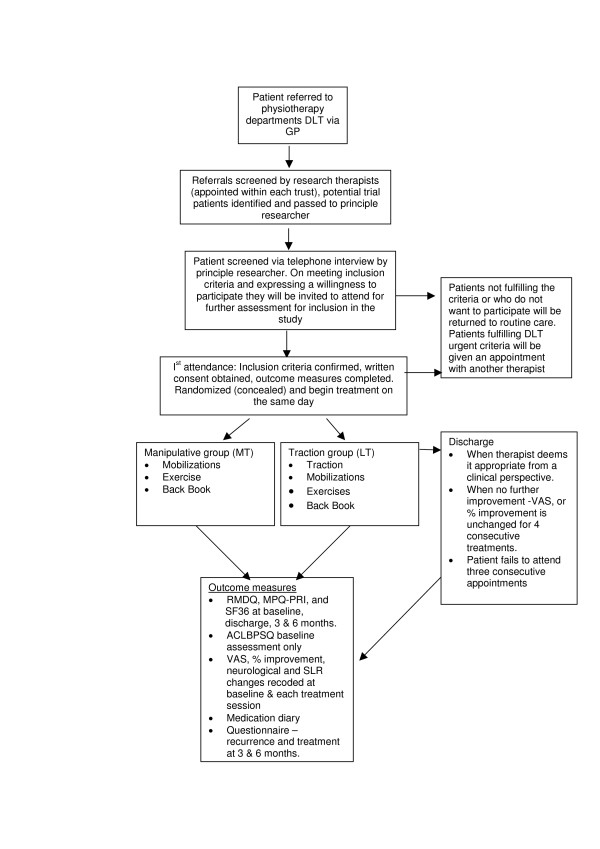
Clinical trial procedure.

The research therapist (who was blind to group allocation), performed the baseline and outcome measures. Randomization was performed by an independent researcher not otherwise involved in the trial through a pre-determined randomization table [14]; group allocation was placed in a sealed opaque envelope and numbered 1 – 30 for each trial patient. Patients and therapists were instructed not to reveal to the research therapist the treatment group to which they had been allocated (Fig 1).

Outcome measures were recorded at baseline, discharge and again at 3 and 6 months post completion of treatment; the 3 and 6 month follow-up were completed by post. Primary outcome measures used were: McGill pain questionnaire (MPQ) [15-17], Roland-Morris disability questionnaire (RMDQ) [18], Short-form 36 (SF36) [19], and the Acute LBP screening questionnaire (ALBPSQ) [20,21]. Secondary outcomes were recorded at each treatment session by the treating physiotherapist: visual analogue scale for back and leg pain (VAS) [22,23] and percentage of overall improvement as perceived by the patient [24]. In addition a medication diary was recorded by the patient throughout treatment, and a general questionnaire pertaining to recurrence, further treatment etc was collected at 3 and 6 months.

### Treatment Groups

#### Manual Therapy Group (MT)

Patients randomly assigned to this group received manual therapy, exercises and advice. In the absence of any specific treatment protocol for the management of 'nerve root' this represented the "best treatment for acute LBP" as designated by the Clinical Standards Advisory Group [7] and the Royal College General Practitioners Guidelines [8]. Treating physiotherapists were limited to using the techniques contained within the protocol, but were free to choose the selection of techniques considered most appropriate for that patient at any point during treatment.

*Manual therapy*: was defined as any mobilization and/or manipulation techniques for the spine described by Maitland [25] or Cyriax [26]. The physiotherapist had freedom of choice of which to use and when, and the spinal regions to which they were applied.

*Exercises *included specific exercises and/or advice to stay active; the former included any exercises that the therapist felt was appropriate for an individual patient e.g. mobilizing exercises, abdominal and back strengthening exercises, extension exercises or core stability.

*Advice: *to stay active included continuing with activities or introducing activity e.g. the addition of short walks, swimming or continuing with reduced activities of daily living; gentle mobilizing or strengthening exercises could also be included. Relevant advice was also given to the patient e.g. sleeping positions, sitting positions, education re prognosis and recurrence rates etc. The "Back Book" [27], an evidence-based patient education booklet, was given to all patients by the treating therapist to reinforce the information verbally given to the patient.

#### Traction group (LT)

This arm of the trial was based upon the same intervention as the manual therapy group but with the addition of motorized (static) lumbar traction. Lumbar traction was used initially with these patients but the therapist could also select to use mobilization techniques in conjunction with traction, or to exchange traction for mobilization techniques as the patient improved (therapist's clinical judgment). The parameters for traction application were established from expert opinion and from the results of a UK wide survey (Table 1).

**Table 1:** Traction parameters: nerve root patients

Traction parameter	Recommended guidelines (Harte et al 2005)
Traction bed	Split table motorized traction unit
Traction Position	Fowler position (hips and knees flexed to 90 supported on stool
Traction Weight	5 – 60 kg
Traction duration	10 – 20 min each session
Traction Frequency	2 – 3 times per week

### Excluded treatments

Patients were not permitted to receive any other types of manual therapy (e.g. Mulligan, McKenzie regimes), electrotherapy, or any additional interventions (acupuncture, taping, corset, heel raises) during the intervention period of the trial. These were excluded as the UK wide survey [3] informing this trial had not indicated a common use of these modalities in conjunction with traction.

### Statistical analysis

All data were scored and entered onto the Statistical Package for the Social Sciences (version 11) for analysis. Due to the small sample size and the chance of Type 2 error, inferential statistics were not used in the analyses; instead descriptive statistics were employed to compare the data between the two groups (median and interquartile range, as data were skewed). Intention to treat analysis was performed. The imputation method used to replace missing data for all study participants who failed to complete treatment or follow up was the last available score forward method; an alternative per protocol was also performed for those that completed treatment and follow up. SF36 was analysed as norm-based data and presented as component scores for physical and mental components. Appropriate descriptive statistics are presented for baseline characteristics (Table 2).

**Table 2:** Baseline demographics for both groups

	LT group (n = 16	MT group (n = 14)
**Age **(Mean, SD)	45.25 (7.99)	42.79 (12.09)
		
**Gender **(%, N)		
Male	37.5 (6)	42.9 (6)
Female	62.5 (10)	57.1 (8)
		
**Employment **(%, N)		
In paid employment	68.8 (11)	78.6 (11)
Not in paid employment	31.5 (5)	21.4 (3)
		
**Job description **(%, N)		
Heavy manual work	25 (4)	35.7 (5)
Light work – desk/supervisory	31.3 (5)	28.6 (4)
Homemaker	1.8 (3)	21.4 (3)
Professional	12.5 (2)	7.1 (1)
Retired	6.3 (1)	7.1 (1)
Incapacity benefit	6.3 (1)	0
		
**Sick leave from work **(%, N)		
Not applicable	31.5 (5)	14.3 (2)
At work	43.8 (7)	42.9 (6)
Off work due to LBP	25 (4)	42.9 (6)
		
**Weeks duration of LBP **(Median, IQR)	6.5 (4.8)	6 (4)
		
**Number of episodes of LBP in the past year **(%, N)		
None	81.3 (13	42.9 (6)
1–3	6.3 (1)	42.9 (6)
Constant LBP	12.5 (2)	14.3 (2)
		
**Number of months since last episode of LBP **(%, N)		
No episodes	81.3 (13)	35.7 (5)
3–6 months	0	35.7 (5)
7–12 months	6.3 (1)	7.1 (1)
>1 year	0	7.1 (1)
Constant LBP	12.5 (2)	14.3 (2)
		
**Previous treatment for LBP **(%, N)		
Yes	12.5 (2)	14.3 (2)
No	50 (8)	50 (8)
Not applicable	37.5 (6)	28.6 (4)
		
**Participation in physical activity **(%, N)	62.5 (10)	35.7 (5)
		
**Continued with activity since onset of this episode **(%, N)	18.6 (3)	21.4 (3)
		
**Smoker **(%, N)		
Current smoker	25 (4)	28.6 (4)
Past smoker	6.3 (1)	21.4 (3)
None smoker	68.8 (11)	50 (7)

**Neurological/Neurodynamic changes **(%, N)		

Reflex – reduced/absent	18.8 (3)	35.7 (5)
Muscle power – reduced/absent	37.5 (6)	50 (7)
Sensation – decreased/hypersensitive	18.8 (3)	28.6 (4)
SLR – restricted and reproducing leg pain	100 (16)	78.6 (11)
Crossed SLR	6.3 (1)	21.4 (3)

**Outcome measures **(Median, IQR)		

RMDQ	10 (4.5)	11.5 (8.5)
MPQ-PRI	20.5 (9)	29 (20)
ALBPSQ	104 (42.5)	112 (49)
SF36 Physical component score (PCS)	31 (3.6)	36.1 (23.4)
SF36 Mental component score (MCS)	46.5 (17.2)	47.3 (21.3)
VAS back pain	5 (4.5)	5 (4.5)
VAS leg pain	7 (3.5)	5.6 (3.5)

## Results

**Figure 2:**
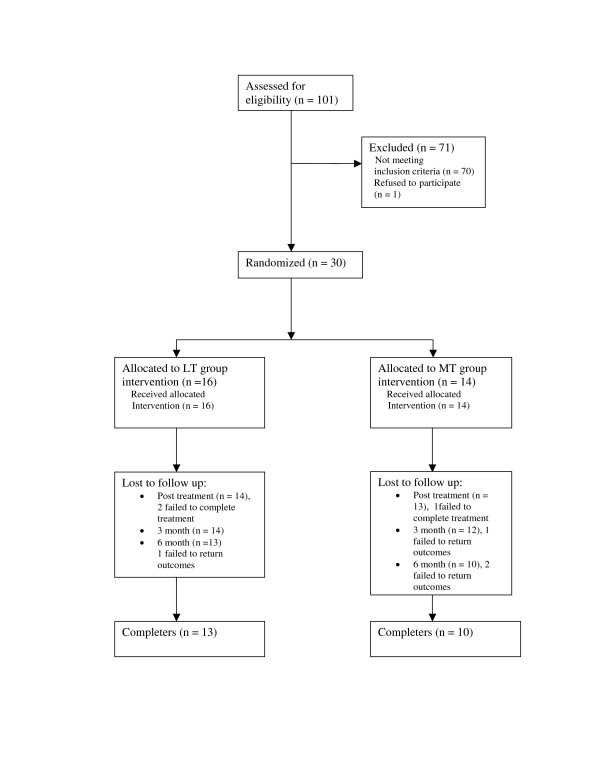
Consort flow diagram.

A total of 101 patients were screened; 30 patients entered the trial between March 2004 and February 2005 (Figure 2 CONSORT flow diagram shows the progression through the trial).

### Compliance with treatment and follow up

All patients received the treatment to which they were allocated; 27 patients completed treatment, 3 patients failed to complete treatment, one patient was lost to follow up at 3 months and a further three patients at 6 months (Fig 2). Subjects in each group received a similar number of treatments (LT, mean 11.4, SD 5; MT mean 10, SD 3.3) lasting no longer than 30 min at each session and were seen for treatment on an average of twice weekly (Mean LT 2.3, SD .79; MT 2; SD .73), ensuring equal contact time for each group.

### Patient demographics

The mean age of patients in the study was 44.1 years (SD 10 years; range 29 – 60 years); 40% (n = 12) were male and 60% (n = 18) were female (Table 2). The mean duration of the current episode of LBP was 7 weeks (SD 2.7 weeks; range 4 – 12 weeks).

The baseline demographics, clinical characteristics, and outcome measure scores showed some differences between groups; most notably was that the MT group had a higher proportion off work due to LBP, a greater history of episodes of LBP, they participated less in physical activity and they also had more neurological signs than the LT group. These findings are possibly reflected in the higher median scores for several of the outcome measures and may have an impact on the results of this study (Table 2).

### Outcomes

**Figure 3:**
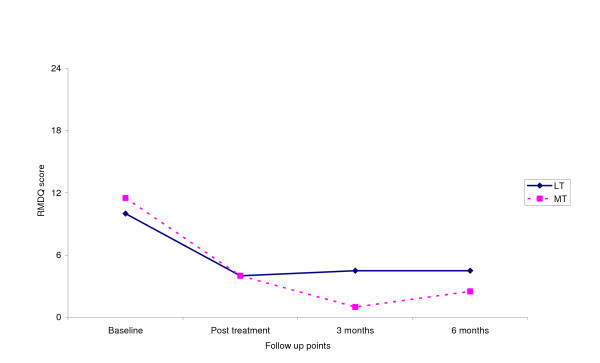
Median score for RMDQ at follow up points.

**Figure 4:**
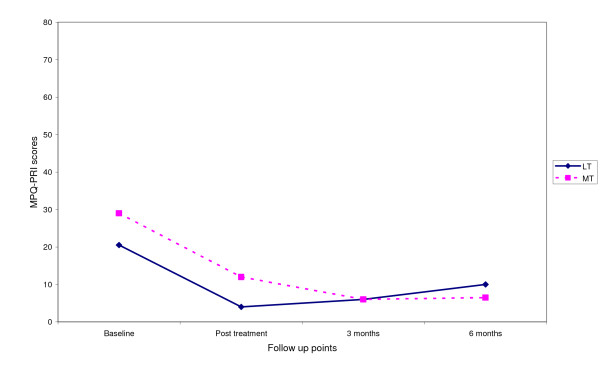
Median score for MPQ – PRI at follow up points.

**Figure 5:**
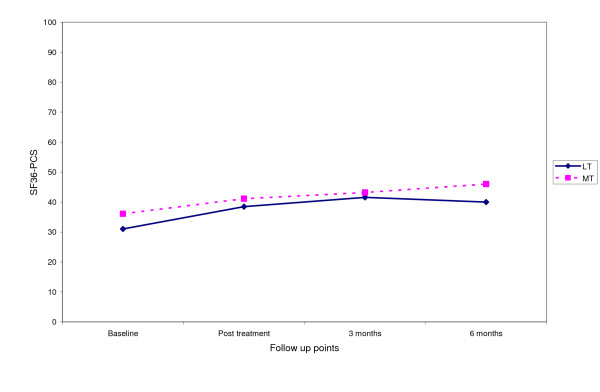
Median score for SF36 physical component score at follow up points.

Fig 3, 4, 5, 6 shows the median points for the main outcomes plotted graphically. These results demonstrate a trend of improvement in both groups at follow up points but there appears to be little difference between the two groups. In comparing the data for those subjects who 'completed' the trial (per protocol) a similar trend was noted with little effect due to the intention to treat analysis (Table 3). Change scores between baseline and the three follow up points demonstrate a similar trend (Table 4).

**Figure 6:**
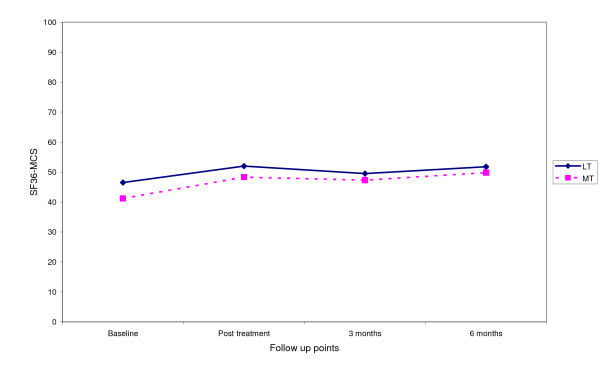
Median score for SF36 mental component score at follow up points.

**Table 3:** Outcome scores at baseline, post treatment, 3 and 6 months follow up for completers and intention to treat analysis

	**Per protocol analysis**		**Intention to treat analysis**	
	
	LT Group (n = 13) Median (IQR)	MT Group (n = 10) Median (IQR)	LT Group (n = 16) Median (IQR)	MT Group (n = 14) Median (IQR)
RMDQ baseline	10 (4.5)	11.5 (8.5)	10 (4.5)	11.5 (8.5)
after	4 (3)	1.5 (9.5)	4 (5.8)	4 (10.3)
3 months	2 (8.5)	1 (11.3)	4.5 (10.8)	1 (10.5)
6 months	4 (9.5)	1.5 (6.8)	4.5 (15.3)	2.5 (14)
MPQ-PRI baseline	20.5 (9)	29 (20)	20.5 (9)	29 (20)
after	2 (7.5)	9 (16.3)	4 (15.3)	12 (16.5)
3 months	4 (11)	1 (23)	6 (16.5)	6 (21)
6 months	5 (16.5)	3 (20.3)	10 (20.5)	6.5 (21)
SF36 PCS baseline	31 (3.6)	36.1 (23.4)	31 (3.6)	36.1 (23.4)
after	43.9 (13.4)	46.2 (24.1)	38.5 (16.2)	41.1 (21.1)
3 months	46.3 (17.9)	45.9 (27.4)	41.6 (18.6)	43.2 (24)
6 months	42.7 (16.7)	50.5 (23)	40 (15)	46 (22)
SF36 MCS baseline	46.5 (17.2)	41.2 (25.8)	46.5 (17.2)	41.2 (25.8)
after	53.2 (19.3)	50.4 (27)	52 (26.1)	48.3 (25.6)
3 months	55.2 (24.2)	49.5 (27.7)	49.5 (25.8)	47.3 (21.3)
6 months	52.6 (18.4)	55 (15)	51.8 (23)	49.8 (19.8)

**Table 4:** Total change scores for outcomes from baseline to follow up points for completers and intention to treat analysis

	**Per protocol analysis**		**Intention to treat analysis**	
	
	LT Group (n = 13) Median (IQR)	MT Group (n = 10) Median (IQR)	LT Group (n = 16) Median (IQR)	MT Group (n = 14) Median (IQR)
Total change score RMDQ				
baseline to post treatment	5.5 (4.5)	3 (5.5)	4.5 (7.3)	3 (8)
baseline to 3 months	6 (7.5)	5 (15.8)	6 (7.8)	5 (15)
baseline to 6 months	7 (7)	5.5 (11.3)	4 (12.3)	3 (12.3)
Total change score MPQ-PRI				
baseline to post treatment	18 (7.8)	16 (27)	17.5 (12.8)	15.5 (25.8)
baseline to 3 months	15.5 (33.8)	15 (33.8)	14.4 (14.3)	15.4 (31.3)
baseline to 6 months	12 (10.5)	19.5 (25.8)	11.5 (17.3)	16.5 (30.8)
Total change score SF36 PCS				
baseline to post treatment	-8.9 (13.9)	-.9 (18.4)	-7.5 (14.6)	-0.5 (13.7)
baseline to 3 months	-15.8 (18.4)	-2.1 (16)	-12.3 (14.2)	-1.6 (17.4)
baseline to 6 months	-10.5 (13.3)	-3.80 (18.8)	-10.2 (10.2)	-4.6 (17.5)
Total change score SF36 MCS				
baseline to post treatment	-4.9 (8.1)	-1 (16.9)	-2.1 (8.5)	-1 (11.9)
baseline to 3 months	-5.7 (18.2)	+1.6 (13.9)	-0.3 (16.7)	+1.6 (13.3)
baseline to 6 months	-7.2 (16.2)	-4.4 (21.6)	-2.3 (15.9)	-2.9 (10.1)

Percentage of overall improvement (as perceived by the patient) at completion of treatment was similar for both groups: LT group median score 90% (IQR 24); MT group 90% (IQR 22.5). Follow up questionnaire at 3 and 6 months showed similar results for both groups (Additional file 1). At the 3 month follow up several patients had sought further treatment: GP (19.2%, n = 5), Orthopaedic Consultant (7.7%, n = 2) and Chiropractor (3.8%, n = 1). One patient had had surgery (discectomy) and two patients were awaiting MRI. At the 6 month follow up two patients were waiting to see an orthopaedic consultant, but despite only small improvements in pain and disability no other treatments were sought.

## Discussion

Recruitment of 'nerve root' patients can be difficult as it has been reported that only 3 – 10% of LBP suffers have these symptoms [4-6]. However this study has demonstrated that a clinical trial with this sub-group of LBP patients is feasible. Recruitment was slow and occurred over an 11 month period with 101 patients screened by telephone to achieve the target of 30 patients for this trial. A larger trial would require a multi-centered design to successfully recruit the numbers required for a fully powered study. The initial telephone screening procedure implemented in this trial was appropriate, and only one patient required attendance for further screening before a descision could be made; furthermore only one patient refused consent to participate in the trial. Although specific data was not retained on those excluded from the trial, the most common reasons were that they did not meet the 'nerve root' criteria or the time from onset of 4–12 weeks. This screening process ensured recruitment of patients with predominantly L4/5 and L5/S1 nerve root symptoms; this is cited as being the most commonly affected nerve root, and thus this group was considered representative [5,28]. This method facilitated the screening of a large number of patients within a minimal timeframe, and would be appropriate for a large multi centre trial.

The results of this study demonstrated an improvement in both groups throughout the trial but with little difference demonstrated between the groups, however this is to be expected in a group of this size. Improvement however cannot be attributed to the intervention as natural improvement can not be discounted as a non-intervention control was not used in this trial. Additionally, while there were differences in baseline characteristics between groups which may have confounded the results a larger randomised trial would overcome this. Analysis was completed through the intention to treat principle (last available score forward method), however on analysis per protocol the results demonstrated similar trends.

As this was a feasibility study with a small sample size and limited statistical power, it may not detect beneficial effects or important relationships between variables therefore a sample size calculation was conducted based on the minimum clinical difference of the RMDQ of 2 points [28]. This indicated 50 subjects would be required for each intervention group (90% power, alpha value of 0.05) and allowing for 15% attrition at three follow-up points a sample size of 76 subjects per group would be required for a larger study [14,29]. This would be achievable in a multi-centered design.

This study was designed to investigate the feasibility of a trial of traction with a clearly defined subgroup of LBP patients, who had previously been identified as the group most likely to receive traction in routine clinical practice [3]. The presence of clinically diverse subgroups of LBP patients may confound broadly based research trials in this area, as particular treatments may only be effective with distinct subgroups of subjects. 'Nerve root' is perhaps the most easily identified and arguably the least disputed subgroup. This study advances previous work on traction with LBP patients with 'nerve root' symptoms in the acute/sub acute phase. A number of previous RCTs have examined the effectiveness of lumbar traction with this group of patients; however many of these failed to define 'nerve root' adequately [30-33], and many have included patients outside the acute/sub acute phase [30,31,33-37]. Only two previous studies adequately defined nerve root involvement and included patients in the acute/sub acute phase [38,39]: both of these were rated as low quality studies [40]: one with positive [38], and one with negative results [39]. However the Larsson [38] study did not employ clinically appropriate treatment parameters, which is an important issue as it can lead to serious performance bias in a trial [40-42]. The current study has attempted to address these issues, by establishing treatment parameters from expert and clinical opinion [3,40], but it is not possible to state that these are the 'best' treatment parameters available as treatment doses are difficult to establish. However these parameters are important in that they are currently being used by those therapists who feel it is an effective treatment for this subgroup and in the absence of 'best' treatment doses it is a reasonable starting point.

Another possible limitation in the design of previous traction studies has been the use of traction in isolation, as physiotherapists and other clinicians tend to work using a polytherapy approach [3,24,43-46]. Physical therapy is characterised by diverse combinations of treatments which often move from passive towards a more active form of treatment in the latter stages of a management programme [43,46]. Indeed therapists in this study tended to use a combination of all the components of the treatment protocol i.e. manual therapy and/or traction, advice and exercise combined in many different ways throughout the treatment program to reflect the needs of their patients. Delitto (2005) has suggested that we have been looking for the 'magic bullet' for LBP management, with explanatory trials subjecting all non-specific LBP patients to the same treatments; he has argued that this will not provide the answers we seek. Instead studies should be planned that reflect the clinical environment [47]. The pragmatic randomised trial design used in the current study allowed the therapists the flexibility of treating patients individually using a polytherapy approach, based upon clinical reasoning, within a wider research protocol. This design allowed investigation of the effects of a clinically relevant multi-modal approach to back pain.

## Conclusion

This study has shown that a high quality trial with patients with lumbo-sacral 'nerve root' involvement is possible. The trial design has addressed important issues: recruitment of a homogenous subgroup of patients (acute/sub acute stage of nerve root irritation/compression), as well as the use of clinically appropriate treatment parameters (treatment length, frequency and weights). The lack of a clear tend in the data is perhaps not unexpected in an underpowered study and further research is required with a fully powered study to ascertain if there is an effect with the addition of traction to the treatment protocol of mobilisations, advice and exercise. The introduction of a control group receiving advice and medication (as the majority of the patients were on medication) would further confirm the effects or non-effects of these interventions on patients with LBP and 'nerve root symptoms'.

## Competing interests

The author(s) declare that they have no competing interests.

## Authors' contributions

AH is the main author of the article and she designed and managed the trial, acting as screener and outcome assessor and was involved in the interpretation and analysis of the data.

DB and JG have made substantial contribution to the concept, design and interpretation of data. They have also been involved in the critical review of the manuscript at all stages of preparation and have given their approval for publication. All authors have read and approved the final manuscript.

## Pre-publication history

The pre-publication history for this paper can be accessed here:



## Supplementary Material

Additional file 1 Results of follow up questionnaire at 3 and 6 months. This table provides the results of the follow up questionnaire at 3 and 6 month follow up points Click here for file
