# Supplementary material for: The effectiveness of motorised lumbar traction in the management of LBP with lumbo sacral nerve root involvement: a feasibility study
Source: BMC Musculoskelet Disord. 2007 Nov 29;8:118. doi: 10.1186/1471-2474-8-118 (PMC2217540; doi:10.1186/1471-2474-8-118)
Supplement: Additional file 1 — Results of follow up questionnaire at 3 and 6 months. This table provides the results of the follow up questionnaire at 3 and 6 month follow up points [file 1471-2474-8-118-S1.doc]

**Results of follow up questionnaire at 3 and 6 months**

|  | **3 month follow up 6 month follow up** | | | | | |
| --- | --- | --- | --- | --- | --- | --- |
| **% (frequency)** | **Whole cohort**  **(%, n)**  **(n = 27)** | **Traction group (%, n)**  **(n = 14)** | **MT group (%, n)**  **(n = 12)** | **Whole cohort**  **(%, n)**  **(n = 23)** | **Traction group (%, n)**  **(n = 13)** | **MT group (%, n)**  **(n = 10)** |
| Episodes of LBP   - No recurrence - Some recurrence - Constant LBP | 42.3 (11)  42.3 (11)  15.4 (4) | 42.9 (6)  50 (7)  7.1 (1) | 41.7(5)  33.3 (4)  25 (3) | 45 (10)  40.8 (9)  13.6 (3) | 30.8 (4)  61.5 (8)  7.7 (1) | 60 (6)  20 (2)  20 (2) |
| **Episodes of leg pain**   - No recurrence - Some recurrence - Constant leg pain | 30.8 (8)  46.1 (12)  23.1 (6) | 28.6 (4)  50 (7)  21.4 (3) | 33.3 (4)  58.4 (7)  25 (3) | 30 (9)  33.3 (10)  13.3 (4) | 30.8 (4)  53.9 (7)  15.4 (2) | 50 (5)  30 (3)  20 (2) |
| **Leg pain at present**   - Yes | 50 (14) | 57.1 (8) | 41.7 (5) | 34.8 (8) | 38.5 (5) | 30 (3) |
| **Sick leave**   - None - 3-21 days - More than 21 days | 62.5 (15)  29.3 (7)  8.4 (2) | 64.3 (9)  28.4 (4)  7.1 (1) | 60 (6)  30 (3)  10 (1) | 65.2 (15)  30.5 (7)  4.3 (1) | 61.5 (8)  38.5 (4) | 70 (7)  20 (2)  10 (1) |
| **Job change due to LBP**   - Yes | 0 | 0 | 0 | 3.3 (1) | 6.3 (1) | 0 |
| **Additional treatment received**   - GP - Orthopaedic consultant - Surgery - Awaiting MRI - Chiropractor | 19.2 (5)  7.7 (2)  3.8 (1)  7.7 (2)  3.8 (1) | 14.3 (2)  7.1 (1)  0   - 1. (1)   0 | 25 (3)  8.3 (1)  8.3 (1)  8.3 (1)  8.3 (1) | 0  8.7 (2)  0  0  0 | 0  7.7 (1)  0  0  0 | 0  1 (10)  0  0  0 |
| **Disabled living allowance**   - Started due to LBP | 3.8 (1) | 7.1 (1) | 0 | 0 | 0 | 0 |
